# Critical Marangoni Number for Disappearance of Striations during Spin-Coating

**DOI:** 10.1021/acs.langmuir.5c02722

**Published:** 2025-08-04

**Authors:** Suguru Shiratori, Koji Tanikawa, Toma Kamon, Mizuho Ueda, Takashi Kuroiwa, Hideaki Nagano, Kenjiro Shimano

**Affiliations:** † Department of Mechanical Systems Engineering, Tokyo City University, 158-8557 Tokyo, Japan; ‡ Department of Applied Chemistry, Tokyo City University, 158-8557 Tokyo, Japan

## Abstract

This study investigated the striations, which arise as spoke-like thickness undulations during the spin-coating process. It was found that the striations, which once appeared, then disappeared when a mixture of epoxy resin and diglyme solvent was used as the coating fluid. The time-dependent wavelength spectrum was obtained by applying short-time Fourier analysis to the optically measured spatiotemporal thickness variation. The wavelength components corresponding to the striations decreased by 3 orders of magnitude before and after the decay. Under the hypothesis that striations are generated by the type of solutal Marangoni-Bénard instability, the disappearance of the striations can be regarded as the result of the Marangoni number falling below the subcritical value for the occurrence of striations. To determine the critical Marangoni number for the disappearance of the instability, the transient Marangoni number *Ma* was predicted by a numerical simulation taking the thickness-thinning due to centrifugal force and solvent evaporation into account, together with the time evolution of the concentration distribution. The critical Marangoni numbers were determined as values of numerically predicted *Ma* corresponding to the time when the experimentally observed striations started to decay, and they were in the range of *Ma*_c_ = 90–160.

## Graphical abstract

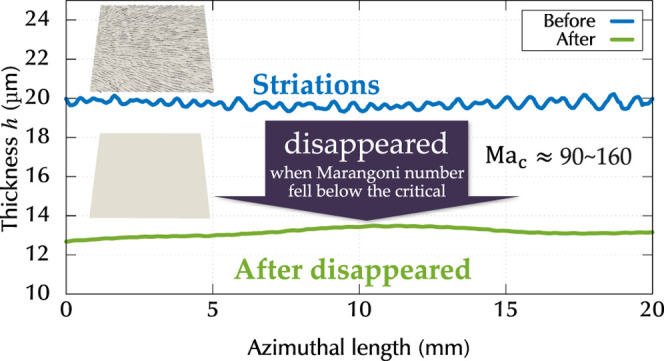

## Introduction

The thin-liquid-film coating process is ubiquitous in a wide range of industrial manufacturing processes, including those for semiconductors, color filters for displays and image sensors, microelectro mechanical systems (MEMS), and antireflection films for optical lenses. For semiconductors, coated photoresist films can often be as thin as the submicrometer range, because they are used as masks for exposure or etching and are then removed after their role is finished. For MEMS devices or color filters, parts of the photoresist films, whose thickness exceeds the order of 10 μm, are not removed and used as the final device structures. Therefore, the film thickness is determined by the design of the device, and its uniformity is strongly required. For general wet coating processes, the photoresist is dispersed and spread on the substrate by rotating the substrate, which is called the spin coating process, and then the film is dried until the solvent has sufficiently evaporated. The thickness of the spin-coated films is nearly uniform, whereas from a local viewpoint, the nonuniform thickness distribution is generated by several physical phenomena during the process.^1,2^ The better-known thickness undulations are “edge-beads” and “striations.” An edge-bead is a ridge of thickness that appears along the substrate periphery. This edge-bead results in a yield loss of product because devices cannot be placed at the region eroded by the edge-bead. The generation of the edge-beads had been investigated by Shiratori and Kubokawa for the case when the bead becomes double-peaked along the radial direction. They proposed a simple explanation for the formation of double-peaked edge-beads.^3^

Striations are another typical thickness undulation that appears as radial spoke-like patterns throughout the film. Striations cause a reduction of the dimensional accuracy and, consequently, the quality of the product. Therefore, it is necessary to investigate how to avoid or suppress striations. The behavior and generation process of striations have been investigated in previous studies.^4–12^ Daniels et al.^6^ proposed a hypothesis for the striation-generation mechanism based on the Marangoni effect.^13^ The cause of the striations was explained by the combination of a Marangoni-Bénard (MB) cell and radially outward flow due to the rotation of the substrate. The MB cell is the fluid dynamical instability driven by the Marangoni effect caused by the surface tension gradient on the liquid surface. The cause of the surface tension gradient is the distribution of either the temperature or the concentration of the surface-active components. Typically, mixtures composed of volatile solvents and an involatile resin are used for the spin-coating process. In the liquid film, the fraction of the resin becomes nonuniform in the thickness direction, and the concentration distribution is generated where the surface side is resin-rich. Under this configuration, disturbances of concentration and velocity can be amplified by the feedback mechanism through the convective mass transfer driven by the Marangoni effect. If this mechanism overcomes diffusion and viscous force, the disturbance exponentially grows and dominates the domain. This hydrodynamic instability is called the Marangoni-Bénard instability and has been studied in previous studies.^14–21^ Although the cause of the MB instability described so far is solutocapillary effect, a similar mechanism holds for the thermal MB instability through the similarity between mass transfer and heat transfer. The intensity of the Marangoni effect can be evaluated by a nondimensional Marangoni number. Pearson solved the linear stability problem for a thermal MB instability,^14^ by defining the Marangoni number as follows:

1 $$Ma^{T}=\frac{\partial \sigma }{\partial T}\frac{T_{*}h}{\mu \alpha }$$

and he reported that the liquid film heated from below becomes unstable when *Ma* ^T^ exceeds a critical condition *Ma*_c_^T^ = 80. In eq 1, ∂σ/∂*T*, μ, and α are the temperature coefficient of surface tension, the viscosity, and thermal diffusivity, respectively. *h* and T_*_ are the thickness of the liquid layer and the characteristic temperature, respectively. For the characteristic temperature, Pearson^14^ employed T_*_ = (∂*T*/∂*z*)*h*, because in his study the constant linear temperature gradient in the thickness direction ∂*T*/∂*z* is assumed. For the case where the temperature distribution differs from the linear profile, a simple definition for T_*_ as the temperature difference between the top and bottom boundaries is applicable because these boundary temperatures can be easily determined.

For the solutal MB instability, the Marangoni number can be defined by replacing the variables of temperature-related with concentration-related, as follows:

2 $$Ma^{S}=\frac{\partial \sigma }{\partial c}\frac{c_{*}h}{\mu D}$$

where ∂σ/∂*c*, *D*, and c_*_ are the concentration coefficient of the surface tension, the diffusivity, and the characteristic concentration, respectively. The critical Marangoni number for the solutal MB instability may differ from that obtained by Pearson,^14^ because the boundary conditions are generally different. For heat transfer problems Dirichlet boundary condition can be applied to either or both the surface and the substrate, whereas for mass transfer problems, the Neumann boundary conditions are applied at both boundaries. Therefore, the critical Marangoni number of the solutal version *Ma* ^S^ may differ from that of the thermal version *Ma* ^T^. Regarding evaluation of the solutal Marangoni number eq 2, the selection of c_*_ is not straightforward because the concentration distribution along the thickness direction cannot be easily known *a priori*, unlike the thermocapillary case. Serpetsi and Yiantsios have investigated the stability of the solutal MB problem for the Schmidt number *Sc* = μ/*ρD* = 1000. They defined the Marangoni number as

3 $$Ma_{c}^{S*}=\Delta \sigma \frac{c_{0}h}{\mu D}$$

where characteristic concentration and concentration coefficient of surface tension are evaluated by the initial concentration c_*_ = *c*_0_ and ∂σ/∂*c* = Δ*σ*, which is the difference between the surface tensions of solvent and resin. The Marangoni instability occurs only if the Marangoni number *Ma* is positive. For the solutal MB problem, *Ma* becomes positive only for the case the volatile solvent has a lower surface tension than that of nonvolatile resin.

The critical Marangoni numbers mentioned thus far are for MB instability without substrate rotation. For the striations that appear during spin-coating, the Marangoni effect is believed to be the dominant mechanism; nevertheless, the Marangoni number has rarely been evaluated. Haas and Birnie have performed spin-coating experiments using spin-on-glass^8^ and mixtures of ethyl acetate and PMMA.^5^ They reported that striations were generated earlier for cases of larger rotation rates. Kozuka et al. have investigated the generation of the striations through *in situ* observations using an optical microscope.^10^ They found that the striations disappeared during spin-coating when the evaporation rate of the solvent is low. This disappearance of the striations could be attributed to the disappearance of the MB instability; nevertheless, the corresponding critical Marangoni number was not evaluated.

Evaluation of the Marangoni number is helpful to find optimal coating conditions that can avoid or suppress the striations. Haas and Birnie^9^ have performed numerical simulations and predicted transient thermal *Ma*. They showed that *Ma* changed by an order of magnitude during spin coating. For the solutal Marangoni numbers, evaluation of transient behavior is difficult because many parameters in eq 2 may be time-dependent during spin coating; the concentration difference Δ*c* is initially zero but may become large, the thickness *h* decreases drastically, and the physical properties strongly depend on concentration *c*, which increases as the solvent evaporates. Therefore, the Marangoni number changes by an order of magnitude during the spin-coating. Shiratori et al. evaluated the transient solutal Marangoni number by combining experiments and numerical simulations.^22^ Based on the optically measured spatiotemporal thickness variations of the striations, they found that short-wavelength components (less than 1 mm) vanished suddenly in the middle of the process. They also performed numerical simulations based on a model in which the thickness decreased owing to centrifugal force and solvent evaporation were considered, together with the time evolution of the concentration distribution in the thickness direction. The sudden disappearance of the short-wavelength component observed in the experiment was associated with a decrease in the Marangoni number to the subcritical value for a certain bifurcation of the flow regimes. The corresponding Marangoni number was evaluated in the range 1000 < *Ma* < 1800. In their experiments, relatively long-wavelength components, approximately λ > 2 mm, remained, and striations were still confirmed after spin coating. Thus, the Marangoni number for the generation and disappearance of striations could be expected to be lower, but this has not been reported in previous research. This study aimed to evaluate the Marangoni number corresponding to the disappearance of striations. To achieve this experimentally, either the growth or decay processes must be observed. However, the former is difficult because the Marangoni number rapidly increases in the early stages of spin coating.^22^ In this study, we attempted to observe the decay process of striations after they were generated. One possible way to realize this is to select a solvent with a moderate vapor pressure. In this study, we selected diglyme as the solvent and confirmed the disappearance of the striations. The setup and results of the experiment are described in Experiment section. In Modeling section, the mathematical formulation and results of numerical simulations are presented. The critical Marangoni number is evaluated in the Critical Marangoni Number section using the results of experiments and numerical simulations. Conclusions are provided in Conclusion section.

## Experiment

### Setup

In all experiments, a coating fluid composed of a solvent-resin mixture was used. This mixture contained diglyme (diethylene glycol dimethyl ether, CAS No. 111-96-6) and epoxy resin (EHPE-3150, CAS No. 244772-00-7, Daicel). The initial concentration of the mixture was adjusted to the desired weight fraction *w*_0_. For each experimental run, 10 cc of the coating fluid was dispensed on a 6-in. silicon wafer with a radius of 75 mm. The coating fluid was then spread by rotating the wafer at a specified rotation rate Ω for a duration of time *T*_s_. The experimental setup is illustrated in Figure 1. The spinning process was performed using a spin coater (MS-A150, Mikasa), and the volume of the liquid dispensed was controlled with a mechanical dispensing buret (NSTP; Nichiryo). After the spinning process was completed, the wafer was placed on a hot plate and the solvent was allowed to dry for several minutes.

**Figure 1:**
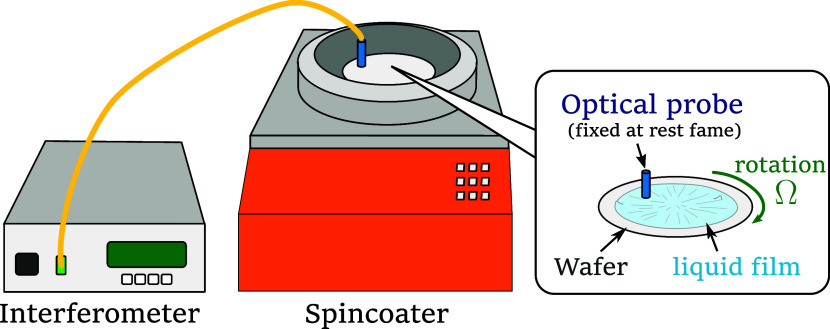
Experimental setup. Spatiotemporal thickness variations along the azimuthal direction were measured by placing the optical probe of an interferometer above the rotating substrate in a spin-coater.

During spin coating, the spatiotemporal variation in film thickness was measured using an interferometer (CHRocodile E, Precitec) equipped with an optical probe (RB200061, Precitec) having a spot diameter of 10 μm. Details of the experimental and measurement conditions are provided in Table 1. The optical probe was fixed in the laboratory frame, while the substrate and the coated film rotated. As a result, the probe traced a circular path in the rotating frame. Consequently, the recorded time series thickness data included both azimuthal distribution and temporal variation. The azimuthal wavelength and its change over time can be analyzed based on the results of time-frequency analysis of the measured thickness data. Azimuthal wavelength λ can be determined as follows:

4 $$\lambda =\frac{2\pi r_{p}\Omega }{f}$$

where *f* is the spatial frequency of the thickness variation in the azimuthal direction. The optical probe was mechanically fixed at a horizontal distance of *r*_p_ = 35 mm from the axis of rotation. Experiments were conducted at two distinct rotation rates: Ω = 50 and 100 rpm Given that the interferometer operated at a sampling rate of 4000 Hz, the minimum resolvable azimuthal wavelength λ_min_ depended on the rotation rate. Specifically, λ_min_ = 91.6 μm for Ω = 50 rpm and λ_min_ = 137 μm for Ω = 75 rpm. If the surface of the film was locally tilted away from the plane perpendicular to the optical axis of the interferometer, the sensor failed to receive adequate reflected light. As a result, the corresponding thickness measurements were unreliable. In this experiment, valid measurements were obtained for surface inclination angles within a range of approximately ± 2–3° During data postprocessing, regions with missing or invalid thickness data were filled in by applying a spline interpolation method.

**Table 1:** Coating and Measurement Conditions for Experiments

item	symbol	value	unit
initial weight fraction	*w*_0_	0.4	
volume dispensed	*V*_0_	10	mL
substrate radius	*R*	75	mm
radial position from rotation axis	*r*_p_	35	mm
rotation rate	Ω	50, 75	rpm
sampling rate	*f*_m_	4000	Hz
max. measurable thickness	*h*_max_	166	μm

### Results

Figure 2a,c show the thickness evolution obtained by the present experiments for the cases where Ω = 50 rpm (a) and Ω = 75 rpm (c). In these figures, time *t* = 0 corresponds to the moment when the rotation began. The thickness data are unavailable for the time interval *t* ≤ 100 s due to two factors. First, the initially dispensed liquid did not extend radially far enough to reach the position of the optical probe, located at a distance *r*_p_ = 35 mm from the center of rotation. As a result, a delay occurred before the liquid arrived at the measurement location. Second, even after the liquid film reached the probe, its thickness exceeded the interferometer’s upper detection limit of 166 μm, preventing valid measurements. The recorded time-series thickness data included information on both azimuthal spatial variations and temporal changes. To analyze how the wavelength evolved over time, a time-frequency analysis based on the short-time Fourier transform (STFT) was applied to the thickness data. In this method, the entire time signal was partitioned into shorter segments of uniform duration. A Fourier transform was then performed individually on each of these segments, allowing the spectral content of each time window to be examined separately. This process provided the Fourier spectrum corresponding to each segment. Figure 2b,d present the time-frequency spectrograms resulting from the application of the SFFT to the thickness variations shown in Figure 2a,c, respectively. In these spectrograms, the horizontal axis corresponds to time *t*. The left vertical axis indicates the frequency normalized by the rotation rate, *f*/Ω, which represents the wavenumber in units of rotation. The right vertical axis shows the corresponding wavelengths λ, calculated from the frequency using eq 4. The color contours indicate the logarithmic intensity of the signal as a function of time and frequency. In a previous study of our research group,^22^ the striation wavelength was approximately λ < 5 mm. In this study, wavelength components longer than 5 mm are not considered. From Figure 2b,d, it can be seen that the dominant components, which seem to correspond to the striations, gradually grow in time ranges 200 < *t* < 600 s for the case of Ω = 50 rpm and 200 < *t* < 450 s for Ω = 75 rpm. During this growth, the wavelength changed from long to short. This change in wavelength could be attributed to the decrease in film thickness because the size of the cells generated by the Marangoni-Bénard instability is known to be proportional to the thickness.

**Figure 2:**
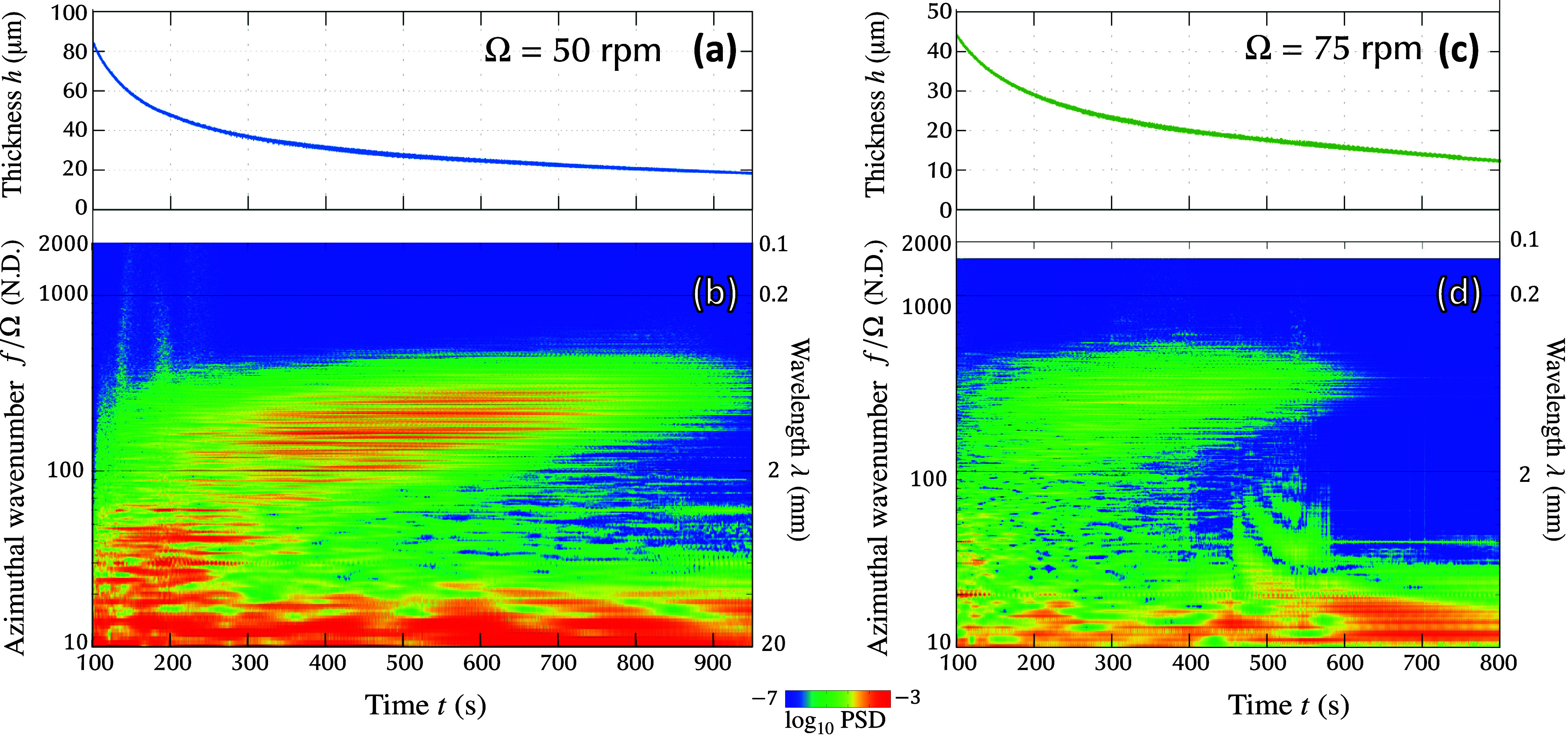
Spatiotemporal thickness variation measured during spin coating of a binary mixture of epoxy resin and diglyme solvent. (a) Time evolution of thickness for the case of Ω = 50 rpm. (b) Spectrogram of thickness variation shown in (a). (c) Time evolution of thickness for the case of Ω = 75 rpm. (d) Spectrogram of thickness variation shown in (c).

Notably, the intensities of these grown wavelength components decayed after attaining their maximum strength. This decay clearly observed in the case of Ω = 75 rpm (Figure 2d), where the power spectrum density for all the components shorter than λ < 5 mm became smaller than 10^–7^. However, for the case of Ω = 50 rpm (Figure 2b), the striation strength remained even at the end of the measurement time range *t* = 950 s. Because the maximum rotation time was limited to 999 s in the spin coater used in this study, data for a further decay time range could not be obtained.

To clearly visualize the wavelength and its dependence on rotation rate Ω, the wavelength spectra at specified time instances are plotted in Figure 3. In this figure, two time instants are selected for each Ω cases. At earlier time instants (*t* = 600 s for Ω = 50 rpm and *t* = 450 s for Ω = 75 rpm), the striation intensity reaches its maximum value. At Ω = 50 rpm, the wavelength component appears from 400 μm to 3 mm with a peak at λ ≈ 1 mm. The peak and wavelength range decreased at rotation rate Ω = 75 rpm. At later instants (*t* = 950 s for Ω = 50 rpm and *t* = 800 s for Ω = 75 rpm) in Figure 3, the intensities of the striation wavelength components were several orders of magnitude lower.

**Figure 3:**
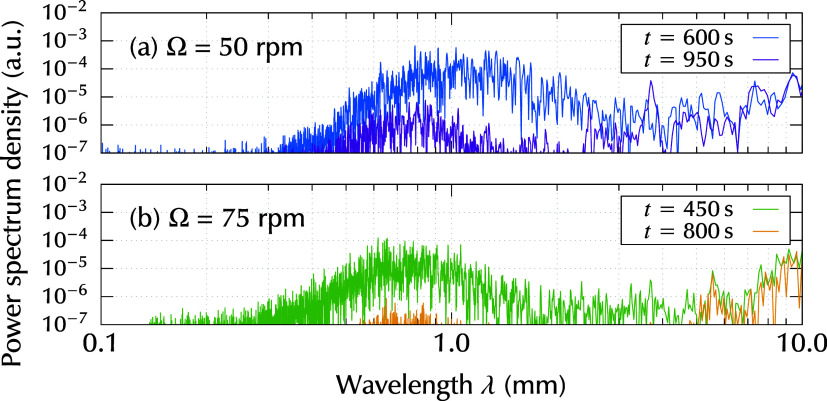
Wavelength spectra at selected time ranges for different rotation rates (a) Ω = 50 rpm and (b) Ω = 75 rpm. For each, two time instants are selected. At earlier time instants, the intensity of the striations reached its maximum. The later time instants are the final phase of the measurement time ranges.

To discuss the decay process quantitatively, the behaviors of selected wavelength components were extracted. Figure 4 shows the time evolution of the power spectrum density for the wavelength components in range 0.5 mm ≤ λ ≤ 2.0 mm. These wavelength values correspond to the wavelengths at which the intensity of the striations reached a maximum. In the later time range shown in Figure 4, it can be clearly observed that the intensity of the striations decayed exponentially, even for the case of Ω = 50 rpm. The red dashed lines in Figure 4 represent the exponential decay function:

$$A\left(t\right)=A_{*}\exp\left(-\frac{t-t_{*}}{\tau_{\mathrm{decay}}}\right)$$

where τ_decay_ is the time constant for the decay. t_*_ and A_*_ are the time and intensity at which the decay starts, respectively. In Figure 4, t_*_ and A_*_ are adjusted differently for each rotation rate Ω. The values of decay time constant τ_decay_ were evaluated as τ_decay_ = 36.9 s for Ω = 50 rpm and τ_decay_ = 33.6 s for Ω = 75 rpm, by considering the linearized decaying behavior as described in the Supporting Information. In Figure 4, the time instants when the striation starts to decay can be evaluated as t_*_ ≈ 750 s when Ω = 50 rpm and t_*_ ≈ 480 s for Ω = 75 rpm. For a larger rotation rate Ω, an earlier t_*_ was confirmed. These time instants t_*_ are used in the discussion in Critical Marangoni number section, where the critical Marangoni number is evaluated.

**Figure 4:**
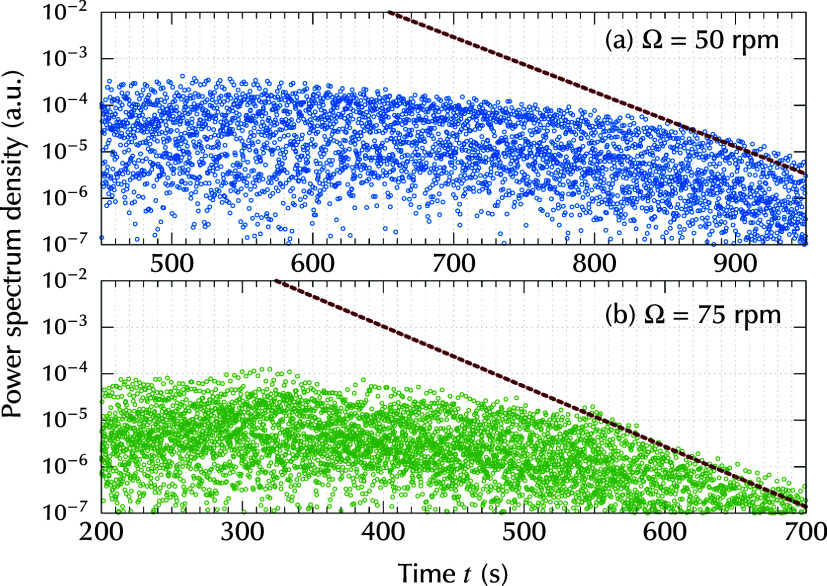
Time evolution of power spectrum density for wavelength components in the range 0.5 mm ≤ λ ≤ 2.0 mm. The red dashed lines show an exponential decay function *A*(*t*) = A_*_ exp­[−(*t* – t_*_)/τ_decay_], where the values for time constant are τ_decay_ = 36.9 s for Ω = 50 rpm and τ_decay_ = 33.6 s for Ω = 75 rpm. A_*_ and t_*_ are properly adjusted for visualization.

## Modeling

### Overview

The previous section provided a quantitative analysis of the disappearance of striations based on optically measured thickness variations. Since the experimental data included only the film thickness, determining the concentration distribution and the physical properties necessary to calculate the Marangoni number was difficult. This section describes how the transient behavior of the solutal Marangoni number was estimated through numerical simulations. The simulation model incorporates the reduction in film thickness caused by centrifugal forces, along with the time-dependent evolution of the concentration profile due to diffusion and evaporation. Although actual liquid films may exhibit radial variations in both concentration and thickness, it is sufficient for evaluating the Marangoni number to consider the average film thickness and the concentration distribution across the film thickness. The dependence of physical properties on concentration is carefully taken into account. The next section outlines how the physical properties of the coating fluid used in this study were measured, and Physical Properties and Evaporation Rate section presents mathematical expressions for their concentration dependence. The governing equations and corresponding boundary conditions are formulated in Governing Equations and Boundary Conditions section, and these equations are further transformed into a time-dependent shrinking coordinate system as explained in Coordinate Transform and Nondimensionalization section. Numerical simulation results, including the time evolution of the Marangoni number, are presented in Numerical Results section.

### Physical Properties and Evaporation Rate

In this model, a binary mixture of a volatile solvent and a nonvolatile resin is considered as the coating fluid. In the experiment, the initial concentration was adjusted in weight fraction *w*. In the remainder of this paper, the volume fraction of resin *c* is used to describe the concentration, unless otherwise stated. The conversion between weight fraction *w* and volume fraction *c* can be written as follows:

5 $$c\left(w\right)=\frac{w/\rho_{r}}{w/\rho_{r}+\left(1-w\right)/\rho_{s}}$$

where ρ_s_ and ρ_r_ denote the densities of solvent and resin, respectively. Regarding physical properties of the mixture, density ρ, surface tension σ, viscosity μ, diffusivity *D*, and evaporation rate *E* were considered to be concentration dependent. The density of the mixture is defined as follows:

6 $$\rho \left(c\right)=\rho_{b}\left[1+\left(c-c_{b}\right)\rho^{*}\right]$$

where ρ_b_ is the density at the reference concentration *c* = *c*_b_:

$$\rho_{b}=\rho_{s}+\left(\rho_{r}-\rho_{s}\right)c_{b}$$

and ρ* is the linear coefficient of ρ for the concentration:

$$\rho^{*}=\frac{\rho_{r}-\rho_{s}}{\rho_{b}}$$

The surface tension of the mixture was measured with the pendant-drop method using DM-501 of KYOWA. Figure 5 shows the measured values of surface tension as a function of resin volume fraction *c*. Here, “°” indicates values for the epoxy-digylme mixture, which were measured in this study, whereas “*” indicates values for the epoxy-xylen mixture, which were drawn from Shiratori and Kubokawa.^3^ The details of the surface tension measurement is described in Supporting Information. In many previous studies, the concentration dependence of surface tension was approximated using a linear function for simplicity. Because this study aimed to evaluate the Marangoni number corresponding to the disappearance of striations, the concentration dependence of the surface tension had to be precisely expressed. In this study, an initial concentration of *w*_0_ = 0.4 was selected, which corresponded to *c*_0_ = 0.35. From Figure 5, it can be seen that the concentration dependence of the surface tension was difficult to express using a linear function, even for the range of 0.3 ≤ *c*. Therefore, we express the surface tension as a power function:

7 $$\sigma \left(c\right)=\sigma_{s}+\left(\sigma_{r}-\sigma_{s}\right)c^{\beta }$$

where σ_s_ and σ_r_ are the surface tensions of the solvent and resin, respectively. β is the power index. The values of σ_s_, σ_r_, and β were fitted using the measured data for all ranges of *c*. The curves are shown as dashed lines in Figure 5. Because the same epoxy resin, EHPE-3150, was used in both mixtures of diglyme and xylene, the surface tension was expected to have the same value in the zero-solvent limit, *c* → 1. Thus, the value of σ_r_ was kept constant during the fitting procedure. The concentration derivative of eq 7, which was required for evaluating the Marangoni number, can be written as follows:

8 $$\sigma_{c}=\frac{\partial \sigma }{\partial c}=\beta \left(\sigma_{r}-\sigma_{s}\right)c^{\beta -1}$$

The surface tension in the epoxy-diglyme mixture exhibited a lower concentration dependence than that in the epoxy-xylene mixture; thus, the Marangoni effect was expected to be smaller. Note, that the solutal Marangoni instability only occurs when σ_c_ is positive.

**Figure 5:**
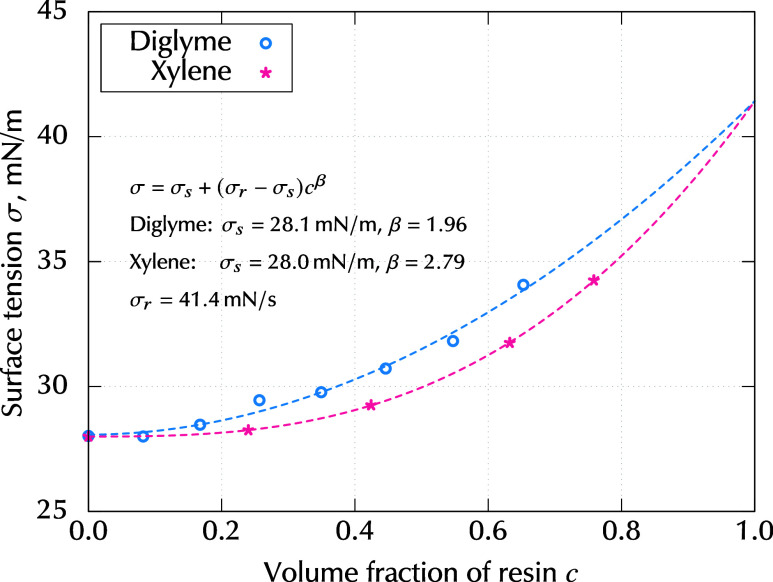
Surface tension of binary mixtures as a function of resin volume fraction *c*, where “°” indicates values for the epoxy-digylme mixture which were measured in this study, whereas “*” indicates values for the epoxy-xylene mixture, which were drawn from Shiratori and Kubokawa.^3^ The dashed lines indicate function σ = σ_s_ + (σ_r_ – σ_s_)*c* ^β^, which was fitted for the entire range of *c*. Fitted values of σ_s_, σ_r_, and β are shown in the figure.

The viscosity of each mixture was measured using a rotational viscometer (LVDV2TCP from Brookfield). Figure 6 shows the measured values of viscosity as a function of resin volume fraction *c*. Here, “°” indicates values for the epoxy-digylme mixture, which were measured in this study, whereas “*” indicates values for the epoxy-xylene mixture, which were drawn from Shiratori and Kubokawa.^3^ The concentration dependence of the viscosity was approximated using the following exponential function:

9 $$\mu \left(c\right)=\mu_{0}\;\exp\left[K_{\mu }\left(c-c_{b}\right)\right]$$

where *K*_μ_ is the concentration exponent, and μ_0_ is the reference viscosity at *c* = *c*_b_. The values of μ_0_ and *K*_μ_ were fitted using the measured data in the limited range *c* ∈ [0.2:1], which was the concentration realized in the experiment. The values derived from the fitted function shown in eq 9 are plotted as dashed lines in Figure 6. In this study, the fluid was assumed to be a Newtonian fluid based on the measurement of shear-rate dependence of the viscosity, provided in the Supporting Information.

**Figure 6:**
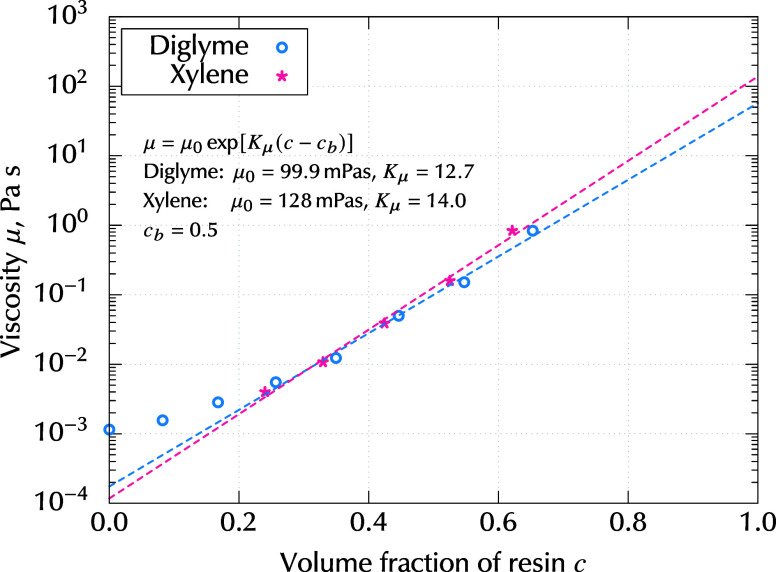
Viscosity of binary mixtures as a function of resin volume fraction *c*. “°” indicates values for the epoxy-diglyme mixture, which were measured in this study, whereas “*” indicates values for the epoxy-xylene mixture, which were drawn from Shiratori and Kubokawa.^3^ The dashed lines indicate function μ = μ_0_ exp­[*K*_μ_(*c* – *c*_b_)], which was fitted for the range *c* ∈ [0.2:1]. Fitted values of μ_0_ and *K*_μ_ are shown in the figure.

In addition to the above-mentioned physical properties, the diffusion coefficient *D* and evaporation rate *E* were important for modeling the coating process. The diffusion coefficient *D* was not directly measured and was assumed to be expressed as follows:

10 $$D\left(c\right)=D_{0}\;\exp\left[-K_{D}\left(c-c_{b}\right)\right]$$

where *D*_0_ is the reference diffusivity, and *K*_D_ is the concentration exponent. Equation 10 is based on the empirical relation that *D* is inversely proportional to the viscosity.^23,24^

The evaporation rate *E* was considered to depend not only on the concentration but also on the rotation rate Ω, as follows:

11 $$E\left(\Omega ,c\right)=E_{0}F\left(\Omega \right){\left(1-c\right)}^{n}$$

where *E*_0_ is a constant in velocity units and F(Ω) is the nondimensional function of the rotation rate. According to previous studies,^4,7,25–27^ F(Ω) was selected as follows:

12 $$F\left(\Omega \right)={\left(1+\frac{\Omega }{\left[\mathrm{rad}{\;s}^{-1}\right]}\right)}^{1/2}$$

Function (1–*c*)^*n*^ in eq 11 is an empirical concentration dependence introduced based on the work done by Eres et al.^28^ The parameters *D*_0_, *K*_D_, *E*_0_, and *n* were determined as described below (for further information, refer to the Supporting Information). In the radially inner region, where the film thickness remains uniform and convective mass transport is negligible, the drying behavior can be described by evaporation and diffusion in the thickness-wise direction. In this area, only the solvent evaporation is the cause of the time change in thickness. Thus, the drying process follows a one-dimensional diffusion model with evaporation boundary conditions. Under this assumption, the parameters *D*_0_, *K*_D_, *E*_0_, and *n* were chosen such that the simulated thickness evolution closely matched the experimental results. Film thickness measurements were conducted using the apparatus detailed in Experiment section. The determined physical property parameters are listed in Table 2.

**Table 2:** Physical Properties of Resin and Solvent

property	symbol	unit	value
density of resin	ρ_r_	kg/m^3^	1171
density of solvent	ρ_s_	kg/m^3^	943.4
reference volume fraction	*c*_b_	N.D.	0.5
surface tension of solvent	σ_s_	N/m	28.1 × 10^–3^
surface tension of resin	σ_r_	N/m	41.4 × 10^–3^
conc. exponent of σ	β	N.D.	1.96
viscosity at *c* = *c*_b_	μ_0_	Pa s	99.9 × 10^–3^
conc. exponent of viscosity	*K*_μ_	N.D.	12.7
diffusivity at *c* = *c*_b_	*D*_0_	m^2^/s	5.0 × 10^–11^
conc. exponent of diffusivity	*K*_D_	N.D.	5.0
evaporation rate at *c* = 0	*E*_0_	m/s	2.2 × 10^–8^
exponent of evaporation rate	*n*	N.D.	0.2

### Governing Equations and Boundary Conditions

This section presents the modeling for the time evolution of the concentration field during spin-coating, taking into account film thinning caused by centrifugal force and solvent evaporation. Since we aimed to predict the transient behavior of the Marangoni number, a one-dimensional profile along the thickness direction was deemed sufficient, under the assumption of uniform film thickness. The process is governed by the time evolution of the mean thickness and a one-dimensional diffusion equation, described as

13a $$\frac{\partial h}{\partial t}=-\frac{2\rho \left(c\right)\Omega^{2}h{\left(t\right)}^{3}}{3\mu \left(c\right)}-E\left(c\right)$$

13b $$\frac{\partial c}{\partial t}=\frac{\partial }{\partial z}\left(D\left(c\right)\frac{\partial c}{\partial z}\right)$$

where *h*(*t*) and *c*(*t*, *z*) are the film thickness and the volume fraction of resin, respectively. *t* is the time, and *z* is the coordinate in the thickness direction with its origin on the substrate. In eq 13, the first and second terms on the right-hand side represent the film-thinning due to the centrifugal force and the solvent evaporation, respectively.

The following boundary conditions were considered:

14a $$\frac{\partial c}{\partial z}=0\;\mathrm{at}\;z=0$$

14b $$D\left(c\right)\frac{\partial c}{\partial z}=E\left(c\right)c\;\mathrm{at}\;z=h\left(t\right)$$

Equations 13 to 14 are not strictly mass conserving formulation, since the density is varying depending on the concentration. The change of the density due to evaporation near the interface may influence the thickness behavior, which is neigther included in Eqs 13 nor [Disp-formula eq14]. This effect is ignored in this study, based on the order estimation described in Supporting Information and the previous study by Diddens et al.^29^

### Coordinate Transform and Nondimensionalization

Because the film thickness decreases as time advances, the spatial domain in the present problem varies, thus, the boundary conditions are difficult to apply. To simplify the problem, the governing equations described in the original (*z*, *t*) coordinates were transformed to shrinking coordinates, (ζ, τ), using the following conversion:

15 $$\zeta =\frac{z}{h\left(t\right)},\tau =t$$

Using the derivatives of the coordinate transforms:

16a $$\frac{\partial \zeta }{\partial z}=\frac{1}{h\left(t\right)},\frac{\partial \tau }{\partial z}=0$$

16b $$\frac{\partial \zeta }{\partial t}=-\frac{\zeta }{h\left(t\right)}\frac{\partial h\left(t\right)}{\partial t},\frac{\partial \tau }{\partial t}=1$$

the diffusion eq eq 14 can be transformed using chain rules as follows:

17 $$\frac{\partial c}{\partial \tau }-\frac{\zeta }{h}\frac{\partial h}{\partial t}\frac{\partial c}{\partial \zeta }=\frac{1}{h^{2}}\frac{\partial }{\partial \zeta }\left(D\frac{\partial c}{\partial \zeta }\right)$$

Using the scales listed in Table 3, the governing equations are nondimensionalized as follows:

18a $$\frac{\partial H}{\partial \tau }=-\frac{\mathrm{TaSc}}{6\mathrm{Sh}}\frac{\mathrm{\rho ̂}H^{3}}{\mathrm{\mu ̂}}-Ê$$

18b $$\frac{\partial c}{\partial \tau }=\frac{\zeta }{H}\frac{\partial H}{\partial \tau }\frac{\partial c}{\partial \zeta }+\frac{1}{\mathrm{Sh}H^{2}}\frac{\partial }{\partial \zeta }\left(\mathrm{D̂}\frac{\partial c}{\partial \zeta }\right)$$

and the nondimensional forms of boundary conditions are

19a $$\frac{\partial c}{\partial \zeta }=0,\;\mathrm{at}\;\zeta =0$$

19b $$\frac{\partial c}{\partial \zeta }=\frac{ÊH}{\mathrm{D̂}}\mathrm{Shc},\;\mathrm{at}\;\zeta =1$$

In eqs 21 and 22, *Ta*, *Sh*, and *Sc* are the nondimensional numbers defined as follows:

20 $$\mathrm{Ta}=\frac{4\rho_{b}^{2}\Omega^{2}h_{0}^{4}}{\mu_{0}^{2}},\mathrm{Sh}=\frac{E_{0}h_{0}}{D_{0}},\mathrm{Sc}=\frac{\mu_{0}}{\rho_{b}D_{0}}$$

The nondimensional forms of the thickness and physical properties are defined as follows:

21 $$H=\frac{h}{h_{0}},\mathrm{\rho ̂}=\frac{\rho }{\rho_{b}},\mathrm{\mu ̂}=\frac{\mu }{\mu_{0}},\mathrm{D̂}=\frac{D}{D_{0}},Ê=\frac{E}{E_{0}}$$

During the nondimensionalization, the characteristic thickness is selected as *h*_0_ = 100 μm.

**Table 3:** Scales of the Dimensionless Variables

	length	time
scale	*h*_0_	*h*_0_/*E*_0_
value	100 μm	4.55 × 10^3^s

For evaluation of the transient Marangoni numbers, the following definition is employed:

22 $$\mathrm{Ma}=\frac{\partial \sigma }{\partial c}\frac{\Delta \mathrm{ch}}{\mu D}$$

where ∂σ/∂*c* is the concentration coefficient of the surface tension, which is concentration-dependent, as defined in eq 8, and Δ*c* is the concentration difference between the surface and bottom:

23 Δc=c(z=h)−c(z=0)

Compared to the eq 2, Δ*c* is selected as the characteristic concentration c_*_. Using the nondimensional numbers, eq 27 can be rewritten as follows:

24 $$\mathrm{Ma}=\frac{\partial \sigma }{\partial c}\frac{h_{0}}{\mu_{0}D_{0}}\cdot \frac{\Delta \mathrm{cH}}{\mathrm{\mu ̂}\mathrm{D̂}}$$

### Numerical Results

The governing equations and boundary conditions eqs 21 to 24 were solved numerically using finite differences. The values of rotation rate were selected as Ω = 50 and 75 rpm, which are the same as those in the experiments. The corresponding Taylor numbers are *Ta* = 1.23 × 10^–2^ and 2.76 × 10^–2^, respectively. The nondimensional numbers and initial conditions used in the present calculation are listed in Table 4.

**Table 4:** Non-Dimensional Numbers and Initial Conditions Used in the Present Numerical Simulations

parameter	symbol	values
Schmidt number	*Sc*	1.89 × 10^6^
Sherwood number	*Sh*	4.40 × 10^–2^
Taylor number	*Ta*	1.23 × 10^–6^,
		2.76 × 10^–6^,
initial concentration	*c*_0_	0.35

The numerical results for the time evolution of the thickness are plotted in Figure 7 by dashed lines, whereas experimental results are plotted by solid lines, for comparison. In the numerical simulation, the initial state was assumed when the dispensed liquid reached the substrate periphery and the liquid film covered all the substrate, whereas *t* = 0 in the experiment corresponds to the time when the rotation began. Thus, the initial time for the numerical results was adjusted so that the differences from the experimental results become minimal. For both rotation rates, the behavior of the thickness decrease calculated by the numerical simulation showed almost complete agreement with the average thickness obtained experimentally. From this agreement between the numerical and experimental results, we can expect that the physical model and applied parameters are sufficient, at least, to express the spatially averaged behavior of the spin-coating liquid film, and the transient Marangoni number can be evaluated based on the present numerical simulations.

**Figure 7:**
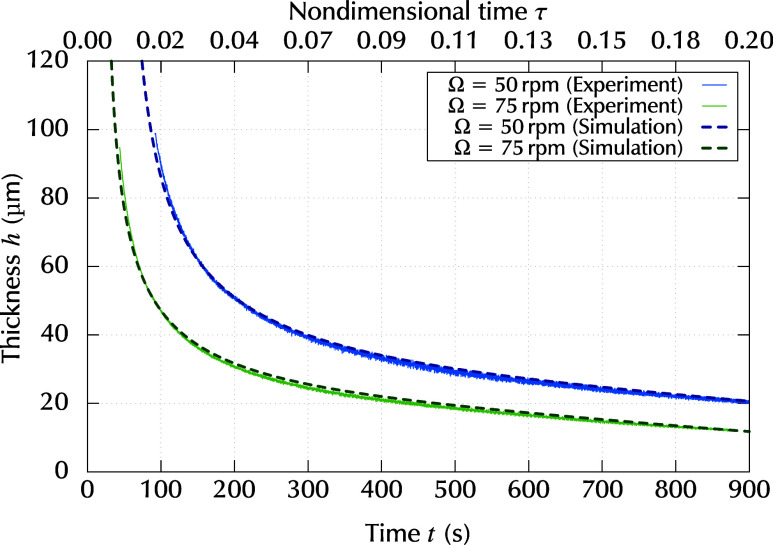
Time evolution of the thickness obtained by the numerical simulations (thick lines) and experimental measurements (thin lines).

Figure 8 shows snapshots of the concentration distribution, *c*(*z*) for the case of Ω = 50 rpm. The solid lines correspond to time instants, and the values of time are indicated by the line colors. In Figure 8, the spatial intervals between adjacent lines become wider for later time ranges. This means that the average concentration increased rapidly in the later time range because the time intervals between the lines were equally spaced. Under the constant evaporation rate, the concentration increases faster when the thickness is small. Therefore, the concentration increased slowly at the beginning of the process and quickly at later stages.

**Figure 8:**
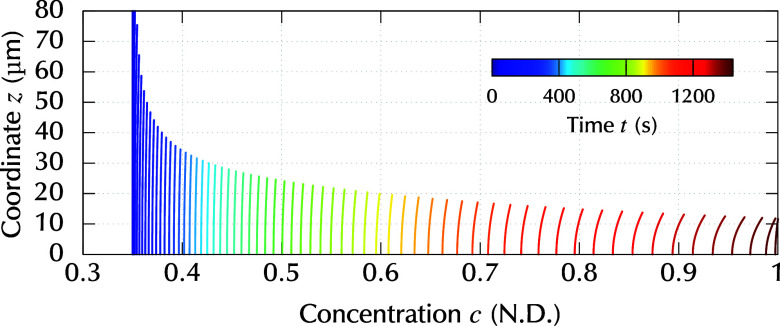
Snapshots of the resin concentration distribution *c*(*z*) with equally spaced time intervals. The rotation rate is Ω = 50 rpm.

To evaluate the transient Marangoni number, the concentration difference, Δ*c*, which was defined as the difference between the top (*z* = *h*) and bottom (*z* = 0), was required and plotted in Figure 9 for the two rotation rates, Ω. From Figure 9, we can observe that the rate of change in Δ*c* was not monotonic. In the early stages of the process, Δ*c* increased rapidly, and then decreased after *t* ≈ 50 s. Subsequently, Δ*c* increased again from the middle stage. This behavior was due to the relationship between diffusivity *D*, evaporation rate *E*, decreasing thickness *h*(*t*), and their concentration dependence. The rate of change in Δ*c* was faster at a higher rotation rate, Ω = 75 rpm. This can be understood in terms of an increase in diffusivity. When the rotation rate was high, the thickness decreased rapidly, the concentration increased, and consequently, the diffusivity decreased rapidly.

**Figure 9:**
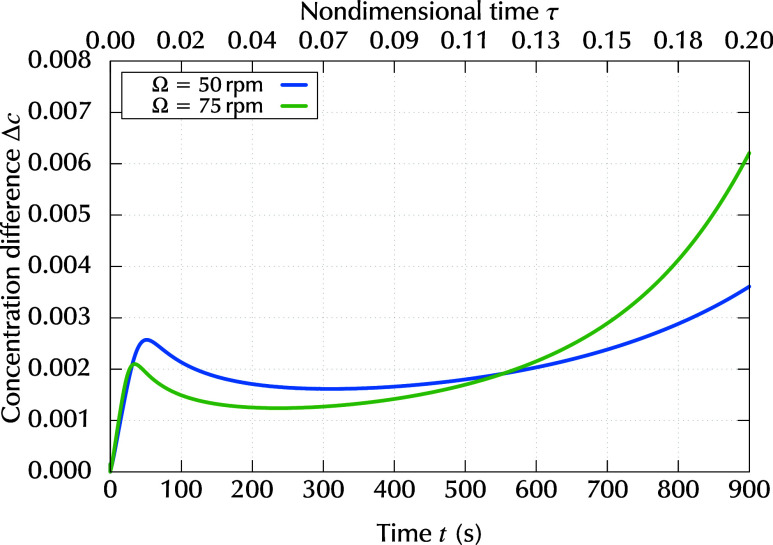
Time evolution of the difference in resin concentration Δ*c* between the top surface (*c*(*z* = *h*)) and bottom boundary (*c*(*z* = 0)) for different rotation rates, Ω.

## Critical Marangoni Number

The transient variation in the Marangoni numbers was evaluated using the above-mentioned results, as shown in Figure 10. In the early stage, the Marangoni number rapidly increased for both rotation rates. This was because of the rapid increase in concentration difference Δ*c* as shown in Figure 9. The Marangoni number began to decrease after reaching its maximum value at *t* ≈ 50–100 s. This decrease was mainly caused by a decrease in thickness. As evaporation advanced, viscosity μ increased, and diffusivity *D* decreased exponentially. In the present study, the exponent *K*_μ_ was larger than *K*_D_. This leads to an increase in the product μ*D*, which appears in the denominator of the Marangoni number. This effect also contributed to a decrease in the Marangoni number at a later stage.

**Figure 10:**
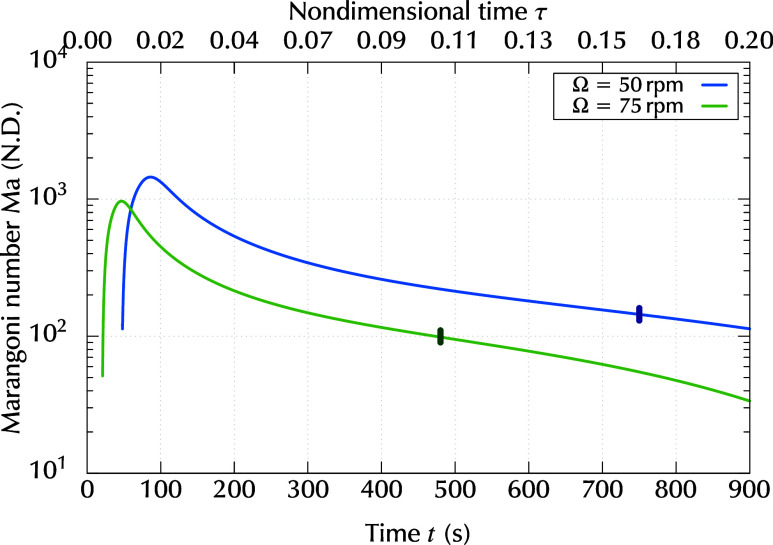
Time evolution of the Marangoni number for different rotation rates Ω. The vertical lines are time instants when the striation started to decay.

The vertical bars in Figure 10 indicate the time instants, t_*_, when the striation started to decay, which were identified from the experiment described in Experiment. The values of time t_*_, corresponding thickness h_*_ = *h*(*t* = t_*_) and Marangoni number *Ma*_c_ are summarized in Table 5. The values were averaged over 5 s. Before the striations began to decay, the wavelength components of the striations maintained their intensity for several hundred seconds, even though the Marangoni numbers were predicted to decrease. This result suggested that the instability mechanism was sustained before *t* < t_*_. This instability was considered to disappear at *t* = t_*_, and no other instability mechanism remained after this time because all of the wavelength components for the striations started to decay and did not remain after a sufficient time. Therefore, the Marangoni numbers corresponding to time *t* = t_*_ could be regarded as the critical values, *Ma*_c_ for the onset of the striations. These Marangoni numbers were dependent on rotation rate Ω and were predicted to be *Ma*_c_ = 90 ± 10 for *Ta* = 1.23 × 10^–6^ and *Ma*_c_ = 145 ± 15 for *Ta* = 2.76 × 10^–6^. These values roughly agreed in order of magnitude with the critical Marangoni numbers for the classical Marangoni-Bénard convection. Specifically, the threshold value of *Ma*_c_ predicted in this study corresponded to the disappearance of striations. Although the critical Marangoni numbers could be different for the onset and offset of striations, the difference between them is, generally, not large.

**Table 5:** Thickness, Time, and Predicted Marangoni Numbers When the Striations Started to Decay

rotation rate Ω	thickness h_*_	time t_*_	predicted Marangoni number *Ma*_c_
50 rpm	22.6 ± 2 μm	750 ± 10 s	145 ± 15
75 rpm	19.4 ± 2 μm	480 ± 10 s	100 ± 10

One noteworthy finding of this study was that the striations could disappear if the Marangoni number became less than the critical value, *Ma*_c_, after they were generated. In practice, whether the striations decayed sufficiently depended on the viscosity, which increased as the solvent evaporated. The time evolution of the viscosity predicted by the numerical simulation is shown in Figure 11. The values of μ corresponding to t_*_ were μ = 1.1 × 10^–1^ Pa s and μ = 6.9 × 10^–2^ Pa s, for Ω = 50 and 75 rpm, respectively. At these viscosity values, the surface deformation of the striations could decay sufficiently within a reasonable time.

**Figure 11:**
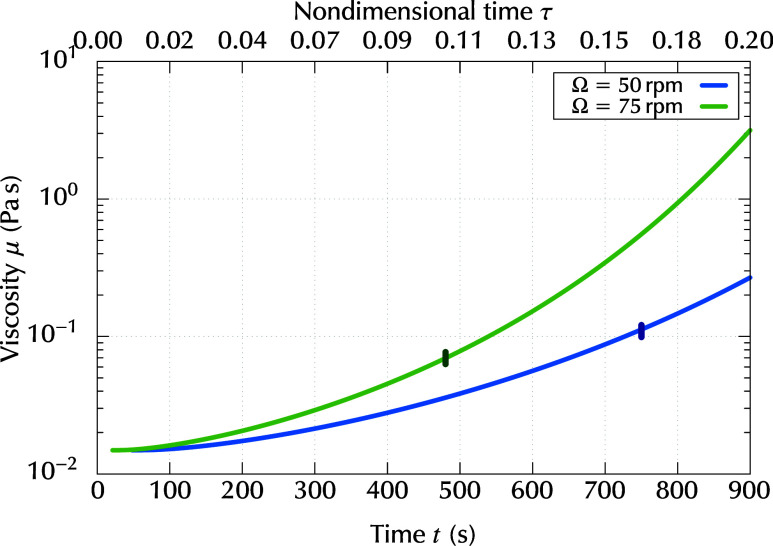
Time evolution of viscosity μ­(*c*) for different rotation rate Ω.

## Conclusions

The formation process for striations, which are radial spoke-like thick undulations arising during the spin-coating of solvent-resin mixtures, was investigated through experiments and numerical simulations. The striations disappeared after they were generated during the spin-coating process when a mixture of diglyme solvent and epoxy resin was used as the coating liquid. The time instances corresponding to the start of the decay of striations were determined using optical measurements of the spatiotemporal thickness variations and short-time Fourier analysis. To predict the Marangoni number, *Ma*_c_ corresponding to the disappearance of striations, a physical model was formulated and numerically calculated. The Marangoni numbers, *Ma*_c_ corresponding to the disappearance of striations were predicted to be *Ma*_c_ = 100 ± 10 for *Ta* = 1.23 × 10^–6^ and *Ma*_c_ = 145 ± 15 for *Ta* = 2.76 × 10^–6^. This study provided the critical Marangoni numbers for striations for the first time. Further investigations are required to determine the dependence of *Ma*_c_ on the rotation rate (Taylor number) or physical properties of the coating liquid. In addition, the physical model used in this study allows for improvement to be more detailed. For effective evaluation of the Marangoni number, the model in this study was highly simplified by dropping the spatial distribution of the thickness and concentration. By considering the Laplace pressure and solutal Marangoni effect, the fully transient simulation and linear stability analysis can be carried out to investigate the instability mechanism behind the striations.

## Supplementary Material



## Notes

Estimation of decay time constant, Model selection, Notes on surface tension measurements, Shear rate dependence of viscosity, and Diffusion coefficient and evaporation rate (PDF)
