# Supplementary material for: Critical Marangoni Number for Disappearance of Striations during Spin-Coating
Source: Langmuir. 2025 Aug 4;41(31):21010–20. doi: 10.1021/acs.langmuir.5c02722 (PMC12356077; doi:10.1021/acs.langmuir.5c02722)
Supplement: Supplementary file 1 [file la5c02722_si_001.pdf]

Supporting Information: Critical Marangoni number for disappearance of striations during spin-coating

Suguru Shiratori,*,† Koji Tanikawa,† Toma Kamon,† Mizuho Ueda,† Takashi Kuroiwa,‡ Hideaki Nagano,† and Kenjiro Shimano†

†*Department of Mechanical Systems Engineering, Tokyo City University, Tokyo, Japan*

‡*Department of Applied Chemistry, Tokyo City University, Tokyo, Japan*

E-mail: sshrator@tcu.ac.jp

Contents

Estimation of decay time constant **S-2**

Model selection **S-4**

Notes on surface tension measurements **S-7**

Shear rate dependence of viscosity **S-8**

Diffusion coefficient and evaporation rate **S-9**

Estimation of decay time constant

In this study, the critical Marangoni numbers are determined based on the time instance when the striations start to decay. In the main body, this decay of the striation is expressed by an exponential function as

$$A(t) = A_* \exp\left(-\frac{t - t_*}{\tau_{\mathrm{decay}}}\right), \tag{S1}$$

where $A(t)$ stands for the amplitude of the thickness undulation. A_* and t_* are the amplitude and time at which the decay starts, respectively. In this section, how the values of the time constant τ_{decay} are evaluated is described.

We consider the decay of the thickness deformation due to the Laplace pressure based on the following 1-dimensional governing equation:

$$\frac{\partial h}{\partial t} = -\frac{\partial}{\partial x}\left(\frac{\sigma h^3}{3\mu}\frac{\partial^3 h}{\partial x^3}\right), \tag{S2}$$

where σ and μ are the surface tension and the viscosity. Here, these physical properties are assumed as constant for simplicity. The spatio-temporal thickness distribution $h(x,t)$ is decomposed by its averaged h_0 and deviation from the average $h'(x,t)$ as:

$$h(x,t) = h_0 + h'(x,t). \tag{S3}$$

The deviation field h' is assumed to be sufficiently smaller than h_0 as $\varepsilon = h'/h_0 \ll 1$. Under this assumption, the governing equation Eq. (S2) can be linearized by neglecting the terms of order $\mathcal{O}\left(\varepsilon^2\right)$ and higher. The linearized equation can be written as:

$$\frac{\partial h'}{\partial t} = -\frac{\sigma h_0^3}{3\mu}\frac{\partial^4 h'}{\partial x^4}. \tag{S4}$$

By applying the Fourier series expansion for h' as:

$$h'(x,t) = \sum_{k=-\infty}^{\infty} h_k(t) \exp\left(\frac{2\pi i x}{\lambda_k}\right), \tag{S5}$$

where h_k is the coefficient for the component of wavelength λ_k, The linearized governing equation can be reduced to the following ordinary differential equation for only a single wavelength λ_k, as:

$$\frac{dh_k}{dt} = -\frac{\sigma h_0^3}{3\mu}\left(\frac{2\pi}{\lambda_k}\right)^4 h_k, \tag{S6}$$

which can be easily solved as:

$$h_k(t) = h_k^0 \exp\left(-\frac{t}{\tau_{\text{decay}}}\right), \qquad \tau_{\text{decay}} = \frac{3\mu\lambda^4}{(2\pi)^4 \sigma h_0^3}, \tag{S7}$$

where h_k^0 and τ_{decay} are the initial amplitude and the time constant, respectively. In the main body of this paper, the decaying process of the thickness distribution is characterized using the time constant τ_{decay}. The detailed values of τ_{decay} are calculated using parameters listed in Table S1. The thickness h_0 is evaluated from the experimental result shown in Fig.2, at the middle stage of the process. Using this thickness value, the corresponding concentration c is evaluated from the result of numerical simulation, and the viscosity μ and the surface tension σ are estimated. The selection of the wavelength λ is sensitive, since the time constant τ_{decay} is proportional to the fourth power of λ. As can be seen from the Fourier spectrum shown in Fig.3, the striations are composed of multiple wavelengths. Although the most strong wavelength components are recognized as $\lambda \approx 1.2\,\text{mm}$ for $\Omega = 50\,\text{rpm}$ and $0.8\,\text{mm}$ for $\Omega = 75\,\text{rpm}$, the further longer wavelength components are involved with sufficient intensity. Since the time constant τ is proportional to the fourth power of the wavelength λ, the short-wavelength components rapidly decay, and the total decaying process is dominated by the long-wavelength components. For the evaluation of the time constant τ_{decay}, we selected the wavelength whose intensity is 1/10 of the peak value.

Table S1: Values used for estimation of decay time constant.

Parameter	Symbol	Units	Value	
			$\Omega = 50\,\text{rpm}$	$\Omega = 75\,\text{rpm}$
Thickness	h_0	µm	23.0	18.0
Wavelength	λ	mm	2.0	1.6
Surface tension	σ	mN/m	31.5	35.1
Viscosity	μ	Pa s	0.459	0.521
Decay time constant	τ_{decay}	s	36.9	33.6

Model selection

Thermal Marangoni effect

In this study, the solutal Marangoni effect is assumed to be the primary driving force of the striation, and the thermal Marangoni effect is neglected in the modeling. In this section, the validity of this assumption is checked by evaluating the thermal Marangoni number, which is defined as:

$$Ma^T = \frac{\partial \sigma}{\partial T} \frac{\Delta T h}{\mu \alpha}, \tag{S8}$$

where $\partial \sigma / \partial T$ is the temperature coefficient of surface tension, ΔT is the temperature difference between film surface ($z = h$) and substrate $z = 0$, h is the thickness, μ and α are viscosity and thermal diffusivity, respectively. The temperature difference ΔT is caused by the latent heat of evaporation. From the energy balance at the interface, the heat flux can be evaluated as:

$$\kappa \frac{\partial T}{\partial z} = -\rho E \mathcal{L}, \tag{S9}$$

where κ is the thermal conductivity of the liquid, $\mathcal{L}$ is the latent heat, ρ and E are the density and evaporation rate, respectively. ρE gives the mass flux across the interface due to the evaporation. Assuming the linear temperature profile in the thickness direction, the temperature difference ΔT can be evaluated as:

$$\Delta T = T_{z=0} - T_{z=h} = \frac{\rho E \mathcal{L} h}{\kappa}, \tag{S10}$$

Values required to evaluate the Marangoni number are summarized in Table S2. The ρ, E are drawn from the main body of this study. The physical properties related to heat transfer c_p and κ, $\mathcal{L}$ are drawn from the technical datasheet of the diglyme (diethylene glycol dimethyl ether), provided by Clariant.[S1] Regarding the temperature coefficient of the surface tension $\partial\sigma/\partial T$, the temperature dependence of the surface tension was measured in this study, for the pure diglyme using the same measurement system explained in the main manuscript. Figure S1 shows the surface tension as a function of the temperature. By using the values listed in Table S2, the thermal Marangoni number Ma^T is estimated as an order of 10^{-1}, which is much smaller than the solutal Marangoni number evaluated in this study. Therefore, the thermal Marangoni effect can be ignored, at least for the case of this solvent.

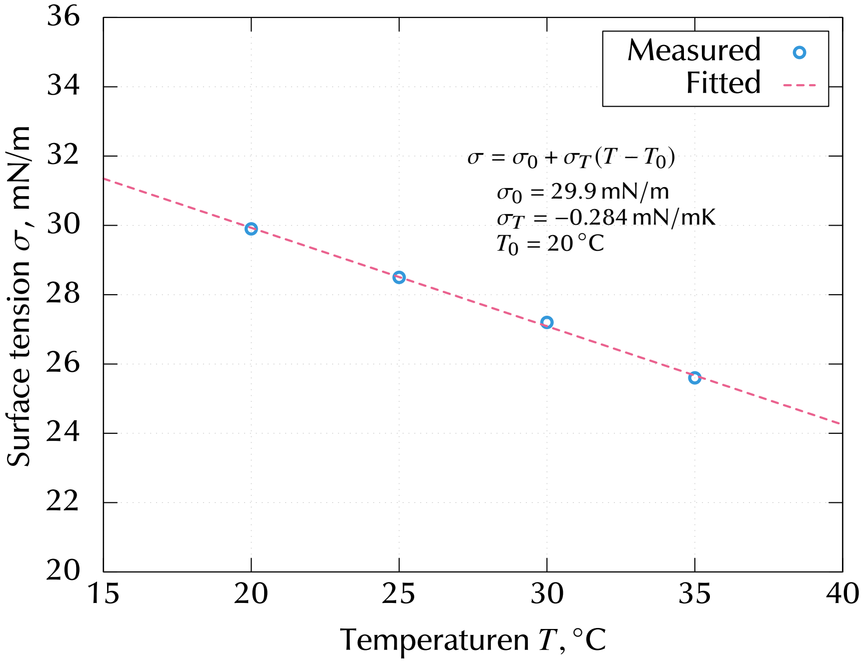

Figure S1: Surface tension of the pure diglyme ($c = 0$) as a function of temperature T. Symbols "○" indicate values measured, whereas the dashed lines indicate function $\sigma(T) = \sigma_0 + \sigma_T(T - T_0)$, which was fitted for the entire range of T. Fitted values of σ_0 and σ_T are shown in the figure.

Table S2: Physical properties and values used for estimation of thermal Marangoni number. The ρ, E are drawn from the main body of this study. The physical properties related to heat transfer c_p and κ, $\mathcal{L}$ are drawn from the technical datasheet of the diglyme (diethylene glycol dimethyl ether), provided by Clariant.[S1] The temperature coefficient of the surface tension $\partial\sigma/\partial T$ is measured in this study, for the pure diglyme using the same measurement system explained in the main manuscript.

Parameter	Symbol	Units	Value	References
Density	ρ	kg/m^3	943.4	Present study
Viscosity	μ	Pa s	1×10^{-2}	Present study
Specific heat	c_p	J/(kgK)	2035	Clariant[S1]
Thermal conductivity	κ	W/(mK)	1.58×10^{-1}	Clariant[S1]
Latent heat of evaporation	$\mathcal{L}$	J/kg	3.1×10^{5}	Clariant[S1]
Evaporation rate	E	m/s	2.2×10^{-8}	Present study
Temp. coefficient of surface tension	$\partial\sigma/\partial T$	N/(mK)	-2.84×10^{-4}	Present study
Thickness	h	µm	100.0	Present study
Thermal Marangoni number	Ma^T	N.D.	1.4×10^{-1}	

Mass conservation

The governing equations used in this study, which are rewritten in the following,

$$\frac{\partial h}{\partial t} = -\frac{2\rho(c)\Omega^2 h(t)^3}{3\mu(c)} - E(c), \tag{S11a}$$

$$\frac{\partial c}{\partial t} = \frac{\partial}{\partial z}\left(D(c)\frac{\partial c}{\partial z}\right), \tag{S11b}$$

are not strictly mass-conserving, since the density varies depending on the concentration. The change of density due to evaporation near the interface may influence the thickness behavior. In this section, the effect of this varying density is evaluated. According to Diddens *et al.*,[S2] the governing equation considering the varying density can be written as:

$$\frac{\partial h}{\partial t} = -\frac{1}{\rho_{z=h}}\left[\frac{\partial(\rho Q)}{\partial x} + \int_0^h \frac{\partial \rho}{\partial t} dz\right] - E(c), \tag{S12}$$

where Q is the flow rate, which corresponds to the centrifugally driven flow rate $2\rho\Omega^2 h^3/3\mu$, in this study. The term $\int_0^h \frac{\partial \rho}{\partial t} dz$ is the effect of the varying density, which is not considered

in this study. The time derivative of density can be evaluated as:

$$\frac{\partial \rho}{\partial t} = \frac{\partial \rho}{\partial c}\frac{\partial c}{\partial t}, \tag{S13}$$

and the integration along z gives

$$\begin{aligned} \int_0^h \frac{\partial \rho}{\partial t} dz &= \frac{\partial \rho}{\partial c}\int_0^h \frac{\partial c}{\partial t} dz, \\ &= (\rho_r - \rho_s)\int_0^h \frac{\partial}{\partial z}\left(D\frac{\partial c}{\partial z}\right) dz, \\ &= (\rho_r - \rho_s)\left[D\frac{\partial c}{\partial z}\right]_0^h, \\ &= (\rho_r - \rho_s)\, Ec. \end{aligned} \tag{S14}$$

Using the values $\rho_r = 1171\,\mathrm{kg/m^3}$, $\rho_s = 943.4\,\mathrm{kg/m^3}$, and $E_0 = 2.2 \times 10^{-8}\,\mathrm{m/s}$, the effect of varying the density term can be evaluated as $\left(\int_0^h \frac{\partial \rho}{\partial t} dz\right)/\rho_{z=h} \approx 5 \times 10^{-9}\,\mathrm{m/s}$. This value is an order of magnitude smaller than the evaporation rate and centrifugal thinning effect. Thus, the effect of the varying density can be neglected in this study.

Notes on surface tension measurements

Here, the detail of the surface tension measurement is described. In this study, the pendant drop method is applied for the measurement. In this method, first, the shape of the drop suspended from a needle is captured by the CCD camera, and then the surface tension is determined by fitting the captured droplet shape with the Young-Laplace equation. In this process, it takes some time for equilibrium where the drop reaches a static shape, and this time becomes longer when the viscosity is large. The waiting time, which is the time difference from dispensing to capturing the drop, can be controlled by the measurement system. In this study, a sufficiently long waiting time has been applied through the preliminary check if the droplet shape becomes static by varying the waiting time. In addition, the radius of

the needle is adjusted depending on the viscosity. The values of waiting time and needle diameter are summarized in Table S3.

Table S3: Detailed conditions for surface tension measumements.

Range of weight fraction w	$0 \sim 0.4$ (0.1 interval)	$0.5 \sim 0.7$ (0.1 interval)
Inner diameter of dispense needle	0.41 mm (22G)	0.84 mm (18G)
Wait time from dispenced to measured	10 s	20 s

Shear rate dependence of viscosity

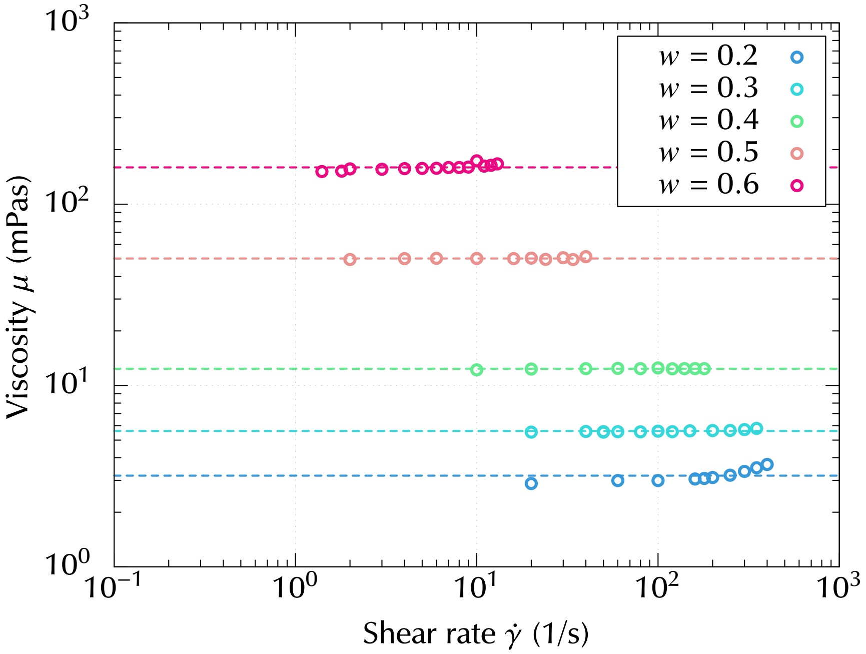

Figure S2: Viscosity μ as a function of shear rate $\dot{\gamma}$, as measured by the cone-plate type viscometer (LVDV2TCP, Brookfield). The fluid was the binary mixture of diglyme (diethylene glycol dimethyl ether, CAS No. 111-96-6) and epoxy resin (EHPE-3150, CAS No. 244772-00-7, Daicel), which was the same as that used in the present experiments. The viscosity was measured for different shear rates and weight fractions, w of the resin. The minimum weight fraction in this measurement was $w = 0.2$ (corresponding to $c = 0.168$), which was much smaller than the initial weight fraction used in the spin-coating experiments. The dashed lines show the fitted constant values. The residuals relative to the fitted constants were less than 1 % for all the weight-fraction cases except $w = 0.2$, which was not used in the experiment.

In the mathematical model employed in this study, the liquid film used for spin coating

was assumed to behave as a Newtonian fluid. Although many resin-solvent mixtures exhibit non-Newtonian viscosity, it was necessary to examine the rheological characteristics of the liquid in question and evaluate whether the Newtonian fluid assumption was valid. Figure S2 presents the viscosity μ as a function of the applied shear rate $\dot{\gamma}$, measured using a cone-plate viscometer (LVDV2TCP, Brookfield). Viscosity measurements were conducted for various shear rates and resin weight fractions w. The dashed lines represent the constant viscosity values obtained by least squares fit to a constant μ. In the spin-coating experiments in this study, the shear rate was estimated to lie within the range of $\mathcal{O}(1 \sim 10)\mathrm{s}^{-1}$. For all tested weight fractions except $w = 0.2$, which was not used in the actual experiments, the deviation from the fitted constants was less than 1 %. Therefore, the influence of shear-rate dependence on viscosity was negligible, validating the use of the Newtonian fluid assumption in this study.

Diffusion coefficient and evaporation rate

In this study, the diffusion coefficient D and the evaporation rate E were identified such that the results of a one-dimensional drying simulation matched the experimental observations. When the centrifugal force is absent, only the solvent evaporation is regarded as the cause of the thickness change. This corresponds to the problem of $Ta = 0$ in the numerical simulation formulated in the **Modeling** section. Provided that the coating fluid is fixed, the parameters governing this process are initial film thickness h_{ini} and the initial concentration c_{ini}. This situation can be realized in the experiments by stopping the substrate rotation when the thickness reaches h_{ini}. Under this situation, the possible differences between numerical simulations and experiments can be considered as the physical properties of diffusivity D and evaporation rate E. In this study, three combinations of the h_{ini} and c_{ini} are selected, and the time evolutions of $h(t)$ were experimentally measured using the optical system described in the **Experiment** section. Then, numerical simulations were carried out by changing

the parameters D_0, K_D, E_0, and n. These values are determined such that the simulated thickness evolution closely matches the experimental results. Figure S3 shows the results of the thickness evolution $h(t)$, where the experimental results are plotted by circles and numerical results are indicated by dashed lines.

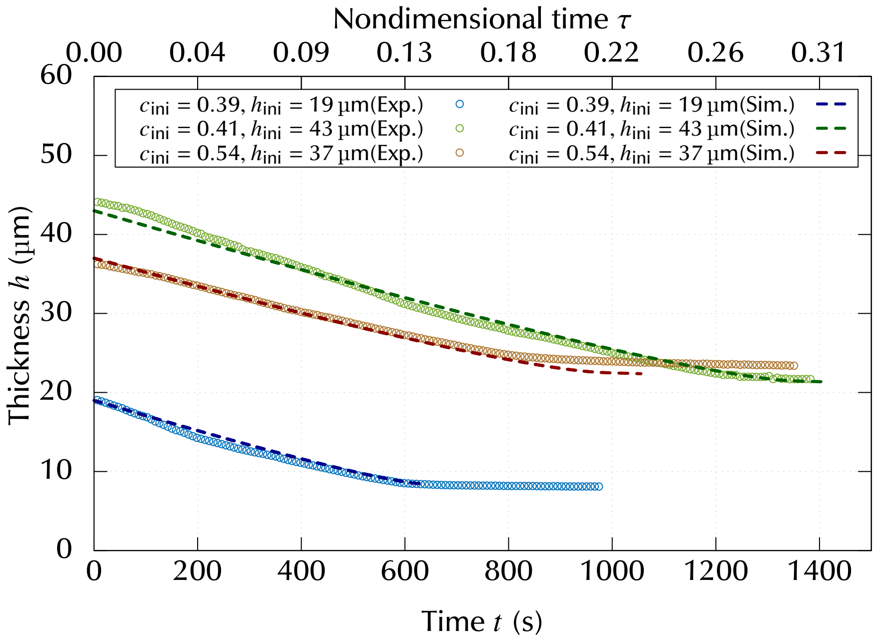

Figure S3: Time evolution of the thickness for the different initial conditions of thickness h_{ini} and concentration c_{ini}. Circles indicate the experimental results, whereas dashed lines are result of numerical simulations.

References

(S1) Diethylene glycol dimethyl ether: Technical Datasheet. 2006.

(S2) Diddens, C.; Kuerten, J.; van der Geld, C.; Wijshoff, H. Modeling the evaporation of sessile multi-component droplets. *J. Colloid Interf. Sci.* **2017**, *487*, 426–436.
